# Expert Consensus to Guide the Classification of Paralympic Swimmers With Vision Impairment: A Delphi Study

**DOI:** 10.3389/fpsyg.2018.01756

**Published:** 2018-10-17

**Authors:** Henrike Joanna Cornelie Ravensbergen, Amarens Doutsen Genee, David Lindsay Mann

**Affiliations:** Department of Human Movement Sciences, Faculty of Behavioural and Movement Sciences, IPC Research and Development Centre for the Classification of Athletes with Vision Impairment, Amsterdam Movement Sciences, Institute for Brain and Behavior, Vrije Universiteit Amsterdam, Amsterdam, Netherlands

**Keywords:** paralympic, classification, vision impairment, swimming, evidence-based

## Abstract

The International Paralympic Committee requires their member sports to develop a classification system that is sport-specific, meaning that the specific ‘class’ in which an athlete competes should be suitable for the degree to which the athlete’s impairment affects performance in that particular sport. However, swimmers with vision impairment (VI) currently compete in classes that were developed on the basis of legal definitions of blindness, failing to consider how vision impacts swimming performance. The aim of this study was to establish expert guidance on the specific requirements for a sport-specific system of classification for VI swimming. A three-round Delphi review was conducted with a panel of 16 people with expertise in VI swimming either as an athlete, coach, administrator, or scientist. There was clear consensus (86%) among the panel that the current classification system used for VI swimming fails to fulfill the aim of minimizing the impact of VI on the outcome of competition. Particularly, the panel agreed that there are a range of aspects of visual function (e.g., depth perception and contrast sensitivity) that are important for optimal swimming performance, yet are not assessed using the current classification system. The panel also identified nine performance components of a swimming race that are mostly likely to be affected by VI. Interestingly, these were spread across all four major segments (start, clean swim, turn, and finish), and weren’t necessarily those performance determinants generally used by performance analysts and coaches. There was also strong agreement that the age at which VI is acquired will substantially impact the ability of a swimmer to reach their full potential in the pool. The main implication is that changes are required to the way that swimmers with VI are classified for para-sport competition. Clear guidance has been provided for how to further the development of an evidence-based classification system.

## Introduction

Fairness is an important virtue in sport. Intuitively, it is unfair if a 50 kg judoka must compete against someone twice their weight, or if a girl needs to sprint a 100 m race against boys of the same age. It is for these reasons that many sports use a system of *classification* to make sport fairer. The goal of a classification system is to systematically reduce the impact of factors such as a competitors weight, gender, or age on the outcome of competition (Tweedy and Vanlandewijck, 2011) in an attempt to legitimize competition. However, the impact of factors such as a person’s weight or age will obviously differ according to the sport being played. For instance, some martial arts and combat sports, such as judo and boxing, group athletes according to their weight to minimize any advantage that a heavier athlete might have. However, greater weight might have little effect, or even be disadvantageous, in other sports such as cycling or climbing. Therefore, the decision about which factors to control for by means of a classification system will clearly depend on the demands of the particular sport, specifically requiring evidence to show that there is an effect of that factor on performance in the sport during competition.

Classification is also used in para-sport. Most commonly, classification is used in para-sports to group athletes into ‘classes’ so that they compete against others who have an impairment that has a comparable impact on sport performance. Specifically, the classification system should account for the impact of the impairment on performance *during competition* (Tweedy and Vanlandewijck, 2011), with performance during competition able to be evaluated either by an overall measurement of performance (e.g., race-time when swimming) or by the measurement of important determinants of performance (e.g., turn time or the ability to propel through the water when in the pool). Historically, classification has been performed in para-sports on the basis of an athlete’s medical diagnosis (e.g., the lesion level of a spinal cord injury, or amputation level). However, this approach typically fails to take into account the impact of the impairment on performance in that actual sport. Just as the impact of age, weight, and gender will differ according to the sport, so too will the impact of an impairment. Therefore, the International Paralympic Committee (IPC) requires all their member sport federations to develop a system of classification that is specific to their sport by relying on evidence that shows how impairment impacts performance in that sport (International Paralympic Committee, 2007; International, 2015b).

In sports organized for athletes with vision impairment (VI), sport-specific classification systems are yet to be developed. While the visual demands during competition are likely to vary considerably across the range of different sports played by people with VI (e.g., swimming, judo, athletics, and skiing), at present the same classification criteria are used for almost all VI sports (with some sports such as Judo choosing to have athletes from all classes compete together). VI sports still rely on their existing medical system that was developed on the basis of the World Health Organization’s definitions of blindness and low vision (World Health Organization, 1989), failing to consider how vision impairment impacts performance in that particular sport. Under the current system, there are three separate classes, where one is meant for athletes who are completely blind or can only distinguish light from dark, one for athletes with severe VIs, and finally one for those with the least severe impairments that still meet the criteria to compete^1^. Our previous Delphi study consulting an expert panel across thirteen VI sports found consensus among the panelists that this existing VI classification system does not achieve the aim to minimize the impact of VI on performance, particularly because of the system is not sport-specific (Ravensbergen et al., 2016).

The need for a sport-specific VI classification system is particularly evident in the sport of swimming given the recent evidence to show that there may be no difference in performance between two of the existing classes. Two studies have compared the performance of swimmers between the three VI classes, with results demonstrating that the race times of the S11 swimmers were significantly longer than those of swimmers in the S12 and S13 classes (Malone et al., 2001; Daly et al., 2009). However, those studies found no significant differences in the race times of swimmers competing in the S12 and S13 classes (Malone et al., 2001; Daly et al., 2009). Similarly, the S11 swimmers took more time to perform their turns when compared to the S12 and S13 swimmers, demonstrating that their poorer vision might specifically influence their ability to execute the turn (Malone et al., 2001; Daly et al., 2009). When compared to Olympic-level swimmers, performance of even the S13 and S12 swimmers was significantly poorer. While these findings are of great interest, there are significant limitations in the usefulness of studies that compare swimming performance across the present sport classes (Tweedy and Vanlandewijck, 2011). In particular, although the results suggest that two VI-classes would be sufficient for VI swimming (i.e., S11 and a combined class for S12 and S13), it could be that there *is* still a need for more than two classes, but that the impact of VI on performance is presently not being evaluated adequately during the classification process. For example, certain swimmers with some remaining sight (e.g., present S12 or S13 class) might still be at a clear disadvantage due to a poorer ability in other aspects of vision (e.g., seeing contrast or movement), but this is not presently detected when testing only visual acuity and visual field during classification.

The critical barrier to the development of a sport-specific system of classification for VI swimming is presently the lack of knowledge about the visual demands of swimming. Tweedy, Mann, and Vanlandewijck have established a five-step research model for the development of sport-specific classification to fulfill the aim of classification to minimize the impact of impairment on the outcome of competition (Tweedy et al., 2016). After identifying the target sport and the impairment to be classified, the second step is to “*develop a theoretical model of the determinants of sport performance”* (Tweedy et al., 2016). To develop a theoretical model, the research needs to identify both the key activities that an athlete needs to perform in their sport of interest, and the factors that most likely determine performance in those key activities (Tweedy et al., 2016). Only once this model has been developed for VI swimming can the next steps in the process be taken, as the outcomes serve as input for the design of experimental studies that assesses the strength of the relationship between each of the aspects of vision and the key determinants of sport performance. Understanding this relationship allows researchers to establish the most appropriate number of sport classes and the boundaries between those classes.

A theoretical model for the determinants of sport performance in VI swimming is still lacking. The VI position stand (Mann and Ravensbergen, 2018) that has been written following expert consultation across VI sports, and adopted by the IPC and the International Blind Sports Federation (IBSA), suggests that consultation with experts (e.g., athletes, coaches, and administrators) is necessary to develop a theoretical model to understand how VI might impact performance in a particular sport. Specifically for swimming, the implication from the position stand is the need to consult experts to canvas opinions on three key items: (1) which aspects of vision impairment impact swimming performance, (2) which components of swimming performance are most likely affected by VI, and (3) under what conditions vision testing for classification should take place (Ravensbergen et al., 2016; Mann and Ravensbergen, 2018). First, it is necessary to establish those aspects of vision that are presently not being tested but may be important for swimming. At present, only visual acuity and visual field are used or classification, however, it might be that other aspects of vision such as contrast sensitivity or movement perception are better at predicting a swimmer’s performance in the pool. For example, the ability to perceive depth might prove to be highly predictive of swimming performance if it helps the swimmer to accurately judge their distance to the wall when timing their turn. Second, the type of actions performed in a swimming race that are likely to be impacted by VI need to be clarified to understand the relationship between VI and swimming performance. VI might not limit a swimmer in their ability to perform every component of the swimming race equally. While vision might be crucial to optimally perform a turn, the ability to react to the sound of the start signal is unlikely to be impacted by VI and so a VI swimmer’s reaction time to the start might not be impacted at all by their impairment. Third, the experts need to be consulted on their opinions about what would be the optimal conditions for testing vision during classification to evaluate the impact of VI on swimming performance. Presently, there are clear rules regulating the conditions under which vision is tested during classification, for example, swimmers are tested while wearing the best optical correction (e.g., glasses). However, there might be a scenario where it is not possible for the swimmer to wear this same optical correction in the pool, in which case the visual conditions during classification could differ markedly to that used when competing in the pool. Finally, expert opinion is required on other issues particular to VI sports that require a sport-specific decision for VI swimming. For example, some sports use blindfolds as a means of equalizing the level of impairment during competition. The choice to use blindfolds (or not) is one that needs to be made by each sport individually. Similarly, sport performance might be impacted by the age at which an athlete acquires their impairment, though it has been established that the nature of this relationship will differ according to the requirements of the sport (Ravensbergen et al., 2016). Expert opinion is desirable to establish whether classification research in VI swimming should take into account the age at which an athlete acquires their VI.

The aim of this study was to establish expert guidance on the specific requirements for a sport-specific system of classification for VI swimming. To do so we particularly focused on reaching expert consensus on (1) which aspects of visual function are most likely to be related to swimming performance, (2) what specific components of the swimming race are most likely to be impacted by VI, (3) how to handle practical and procedural issues when testing vision during classification for VI swimming, and (4) other issues particular to VI sports that require sport-specific decisions.

## Materials and Methods

### Participants

A panel was established that comprised a total of 16 persons considered to possess expertise in VI swimming. Panelists were required to possess specific expertise in VI swimming as (i) an athlete, (ii) a coach, (iii) an administrator, or (iv) a scientist (see **Table 1**). We chose to invite 16 panelists on the basis of our previous experience with the Delphi review process (Ravensbergen et al., 2016). Our previous review consisted of 25 experts, though across all VI sports, suggesting that a lower number was likely to be sufficient when canvassing only one sport. The International Federation that governs para-swimming (*World Para Swimming*) was consulted to identify appropriate panelists who they considered to possess expertise in one of the qualifying categories. World Para Swimming provided an extensive list of potential candidates to serve on the panel, from which a selection was made that maximized representation across the different roles in the sport, and from as many countries as possible. All panelists were required to possess a good level of competency in English. The panelists provided, in accordance with the Declaration of Helsinki, informed consent to participate in the study, with approval for the study granted by the research ethics committee of the Faculty of Behavioural and Movement Sciences at the Vrije Universiteit Amsterdam.

**Table 1:** Characteristics of the panelists.

	N (%)
**Sex**	
Male	12 (75)
Female	4 (25)
**Continent**	
Africa	1 (6)
Asia	3 (19)
Australasia	2 (12)
Europe	5 (31)
North-America	3 (19)
South-America	2 (13)
**Role within VI sport^∗^**	
Administrator	2 (13)
Athlete	6 (38)
Coach	7 (44)
Scientist	1 (6)
Other^∗∗^	1 (6)
**Years of experience in VI swimming**	
0–5	2 (13)
6–10	4 (25)
11–15	5 (31)
**>15**	5 (31)

*^∗^More than one answer was possible. Percentage is based on the number of individuals, not answers. ^∗∗^This panelist was a performance manager, responsible for para swimming within their national federation.*

### Procedure

The study was designed as a Delphi review, which is a structured, systematic method to gather opinions from a panel of experts and to reach consensus on topics of interest (Turoff and Linstone, 1975; Hasson et al., 2000). Over a period of 6 months, panelists independently responded to questions posed to them in each of three rounds of web-based surveys (Qualtrics Research Suite, Qualtrics, Provo, UT, United States). The first survey was designed to question the current VI classification procedures, address the main questions for the development of a sport-specific classification system (identification of *i.* aspects of vision that impact swimming performance and *ii.* components of the swimming race that are likely impacted by VI), and address the major issues identified within our previous Delphi study across all VI sports specifically within swimming (Ravensbergen et al., 2016).

In the second and third round, each section of the survey started with a brief summary of the outcomes from the previous survey and a list of main comments from the panel. The Delphi method is often used to reach *consensus* across the group of experts, with a particular proportion of the panel required to agree on a statement to reach consensus. In this study, the panelists were generally given the option to either agree or disagree with the statement, or to respond that did not feel qualified to answer that specific question. Following their choice, they were always provided the opportunity to provide the rationale behind their answer, which offered insights into the considerations and arguments of the panel. Providing the panelists with this third option ensured that only responses by individuals who felt confident they were knowledgeable in the matter were considered to determine consensus. For each question, the panelists who responded they did not feel qualified to answer that question were excluded for the calculation of the level of consensus. In this study, a minimum of 75% of panelists were required to agree on a statement to reach consensus. This equates to 12 out of the 16 panelists required to agree on a statement. This threshold is at the higher end of the range of consensus levels (i.e., varying from 51 up to 80%) used across many Delphi studies (Hasson et al., 2000).

Panelists were given 3 weeks to complete each survey. All responses were then analyzed and used to prepare the next survey, resulting in a delay of approximately 7 weeks between each round. If the panel reached consensus on a particular issue, the discussion on that topic finished and no further questions were asked in subsequent surveys. If the panel did not reach agreement, the question was clarified and/or rephrased in the next survey based on the comments made by the panelists. When a topic required further clarification, additional questions were posed in the next survey to further explore the panel’s thoughts on the issue.

## Results

All 16 panelists completed the first survey, 14 completed the second survey, and 15 completed the final survey. Each survey was subdivided into nine sections, each covering a specific issue deemed important to classification in VI swimming. The structure of the results section below follows these sections. The process of reaching consensus on the central questions in each section is summarized in **Supplementary Table 1**. The final questions within each section along with panel responses can be found in **Supplementary Table 2**.

### Section 1: Aim of Classification

The aim of classification in para-sport is ‘to minimize the impact of eligible types of impairment on the outcome of competition’ (Tweedy and Vanlandewijck, 2011). Consensus was reached after the first survey (86% of responses; **Supplementary Table 2**) that the current VI classification process used for VI swimmers does *not* entirely fulfill the aim of classification. The majority of panelists (53%) believed that the aim is only partially fulfilled, while 33% believe it is not at all fulfilled. This outcome provides strong support for the need for modifications to the classification system presently used for VI swimming. Problems with the current system frequently mentioned were (1) the system is not specific for swimming, (2) there are broad ranges of impairment within one sport class, (3) visual function tests used are subjective and vulnerable to intentional misrepresentation, and (4) the age at which the impairment was acquired is not accounted for. These concerns were elaborated on within subsequent sections of the Delphi review.

### Section 2: Minimum Impairment Criteria

The *minimum impairment criteria* is the minimum level of impairment required to take part in competition, and by definition should represent the minimum level of impairment that has a significant and adverse impact on performance in that sport (International Paralympic Committee, 2015a). There was consensus within the panel reached after the first survey round (83%) that the current minimum impairment criteria for visual acuity and visual field are independently appropriate for the purposes of VI swimming, that is, that they represent the minimum level of VI that negatively affects swimming performance.

### Section 3: Sport Classes

If an athlete meets the minimum impairment criteria then the next step in the classification process is to allocate the athlete to an appropriate sport class. In VI swimming, there are currently three classes, however, it remains unclear whether these classes provide the most appropriate way of fairly separating VI swimmers into classes.

There was consensus after the first survey round (100%) that swimmers in the S11 class have a significant disadvantage in swimming performance when compared to those in the S12 class. Moreover, the panel reached consensus after the first survey round (80%) that the range of impairments *within* the S11 class have a comparable impact on swimming performance. The S11 class boundary is therefore seen as appropriate to separate the swimmers who are (nearly) blind from the swimmers with more remaining vision.

Even after the third survey the panel could not reach consensus (69% agreed) that S12 swimmers are at a disadvantage when they would compete against swimmers in the S13 class. Additionally, there was no consensus on whether the impact of different levels of impairment on swimming performance is similar *within* either the S12 or the S13 class (**Supplementary Table 2**). In the first and second survey, panelists commented that other factors like the age at which the impairment was acquired might create (dis)advantages across athletes who compete within a single class. In the third survey, we asked panelists to ignore this and assume the hypothetical scenario in which only athletes with acquired impairments were competing. Legitimate class criteria ensure that impairments within one class have a similar impact on performance and impairments across classes significantly differ in their impact on performance. There was in our study no clear support for the current impairment criteria for the S12 and S13 classes as means of separating VI swimmers into appropriate classes.

To further explore the experts’ opinions on the issue of different sport classes in VI swimming, we asked them about their thoughts on what would be the most appropriate number of classes. There was no consensus after the third survey whether a system of *three* sport classes for VI represents the most appropriate number of classes to fulfill the aim of classification. Yet, there was consensus after the third survey (75%) that the range of severities of VI in each sport class should be minimized to ensure equal competition. Classification research investigating the impairment performance relationship is required to determine the most appropriate number of classes, and suitable criteria to separate these classes to be able to fulfill the aim of classification.

### Section 4: Measures of Visual Function

The present VI classification system relies only on measures of visual acuity and visual field to determine a person’s eligibility to compete and for their class allocation. However, this system may fail to account for other aspects of visual function that are likely to be important for sport performance (e.g., contrast sensitivity, or the ability to see movement). The panel reached consensus after the first survey that both visual acuity (79%) and visual field (94%) are appropriate measures to assess the impact of VI on performance in swimming. However, there was also consensus (93%) after the first survey that assessing *only* visual acuity and visual field is *not* sufficient to describe the effect of VI on swimming performance. From an extensive list (**Supplementary Table 3**) of aspects of visual function that was put forward and defined by the authors, the panel after the second survey prioritized the need for tests of depth perception (92%) and light sensitivity (71%) to be considered for use in VI classification (**Table 2**). The majority of panelists (67%) also felt that contrast sensitivity and motion perception were important enough to include in VI classification, however, the set threshold consensus level was not reached on the usefulness of these tests. The panel did not reach consensus that any of the listed aspect of visual function should *not* be included in a future classification assessment.

**Table 2:** Additional measures of visual function considered for inclusion in classification.

Measures of visual function	Important enough to include in VI classification	Not important enough to included in VI classification
Depth perception	**92%**	**8%**
Light sensitivity	**71%**	**29%**
Contrast sensitivity	**67%**	**33%**
Motion perception	**67%**	**33%**
Dynamic visual acuity	58%	42%
Ocular stability	50%	50%
Ocular coordination	44%	56%
Color vision	42%	58%

*The measures highlighted in bold text represent those that a two-third majority of the panel agreed on were important enough to include in VI classification.*

Experimental research investigating the relationship between these different aspects of visual function and swimming performance is required to provide empirical support for which of these measures should be included in the assessment of swimmers with VI in the future.

### Section 5: Procedures for Testing Visual Function During Classification

Currently, those responsible for performing classification (‘classifiers’) evaluate the vision of swimmers by testing each eye independently and allocating a sport class on the basis of the results from the athlete’s *best eye* (i.e., that with the least impairment). The panel fell just short of reaching consensus (71%) after the third survey that the use of the best eye alone represents an inappropriate approach for classification. Instead, the panel’s suggestion was that sport classes should be allocated using the test results when testing both eyes together, because this represents the habitual visual function most likely used when swimmers compete in the pool, and the level that was considered the best vision for optimal swimming performance.

Also, during classification the athlete’s vision is assessed while wearing their best possible correction (e.g., glasses or contact lenses). The reason for this is that this is the best level of vision that is possible for this person, yet it may be that some athletes are unable to wear that correction while in the pool and so would have a level of vision in the pool that is worse than what they were classified with. Our panel reached consensus (83%) after the second survey that classification should continue to take place with the best possible correction in place, irrespective of whether it can be worn in the pool. The main rationale of panelists was that a swimmer can still use their best possible correction during training, for example to learn optimal swimming technique using video recordings or observation.

### Section 6: Impact of VI on Swimming Performance

To know which specific components of a swimming race are likely to be affected by VI is of great value for designing experimental studies that can investigate the impairment performance relationship in detail. In the first survey, we presented the panel with a list of components of a swimming race including a brief description of each. Panelists were then asked to list any all components of the swimming race that they believed were (1) negatively impacted by VI, (2) *not* impacted by VI, or (3) might improve due to VI. It was explained that panelists could use any of the terms from the list, or they could suggest others. In the second survey, we supplemented the list of race components with every component that was mentioned by two or more panelists in their response to the first survey. Since we also asked about aspects that they believed were not affected by VI or ones that might even be improved by VI, the final list provided a clear overview of all race components, not only those that might be affected by impairments in vision. This allowed us to ask the panel to consider each component of the swimming race that could possibly be affected by VI. In the second survey, panelists were asked to provide their opinion about whether they believed that performance on each of these race components would or would not be negatively affected by VI (**Table 3**). In the third survey, we only provided the panel with the outcomes of the second survey and sought no further clarification on this topic.

**Table 3:** Panel ratings of the likelihood that specific components of a swimming race would be negatively impacted by vision impairment.

Swimming race components	Negatively affected	*Not* negatively affected
**Deciding when to initiate the turn**	**93%**	**7%**
**Monitoring the position of competitors**	**93%**	**7%**
**Navigation within the lane**	**86%**	**14%**
**Timing of the final stroke**	**86%**	**14%**
**Maintaining a high speed into the finish**	**86%**	**14%**
**Taking advantage of the allowed length of the underwater phase (after start)**	**79%**	**21%**
**Taking advantage of the allowed length of the underwater phase (after turn)**	**77%**	**23%**
**Direction of the dive**	**71%**	**29%**
**Maintaining a high speed through the turn**	**71%**	**29%**
Maintaining a high speed	57%	43%
Staying streamlined underwater	54%	46%
Dive off the blocks (before hitting the surface)	50%	50%
The act of pushing off the wall	50%	50%
Stroke length	29%	71%
Stroke rate	14%	86%
Reaction time to the start signal	7%	93%

*The components that the panel agreed would be negatively impacted by vision impairment are presented in bold. Underlining indicates components that the panel agreed would not be affected by vision impairment.*

**FIGURE 1:**
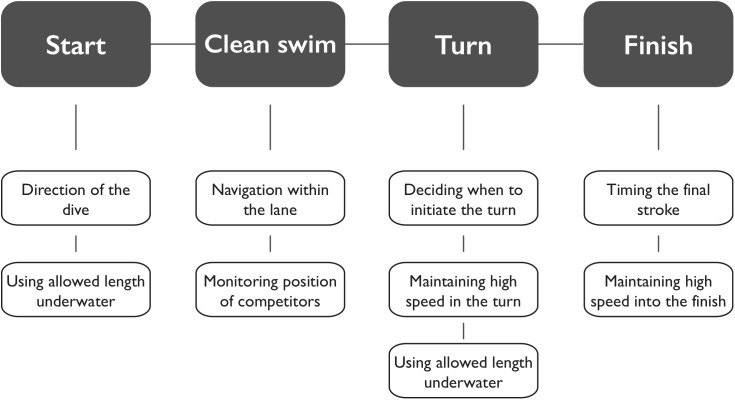
Schematic of the components of a swimming race that the panel agreed are negatively affected by vision impairment.

From those nine components of swimming performance for which the panel agreed would be negatively impacted by VI, a schematic model comprising four major segments was created (start, clean swim, turn, and finish) to represent those aspects of swimming which would be expected to be impacted by VI (**Figure 1**). After both the start and the turn the panel believed that VI swimmers would find it more difficult to stay in their own lane and therefore not use the allowed length (i.e., 15 m) of the underwater phase. The panel commented that this was particularly the case for swimmers who are completely blind; those swimmers often rise to the surface much earlier than what is allowed (i.e., at a distance less than 15 m from the wall). They also agreed that maintaining a high speed into the turn or finish was more difficult for VI swimmers as they might not be able to adequately estimate their distance to the wall. Similarly, their impaired capacity to estimate their distance to the wall was also thought to limit their ability to optimally time their turn and finish. Finally, even during the clean swim the panel felt that VI limits the swimmer’s ability to perform optimally, particularly because their ability to navigate the perfect line through the middle of the lane as well as the ability to monitor the position of their competitors is compromised.

### Section 7: Impact of VI on Different Swim Strokes and Distances

Swimming is a sport with many separate events including four different strokes (freestyle, butterfly, backstroke, and breaststroke) that can be performed over a number of different distances. Athletes presently compete in the same class irrespective of the swimming event they compete in, however it remains unknown whether the impact of impairment would be the same for each of the swim strokes and distances they are performed over.

In general, the panel agreed that the different swim strokes and distances should not require separate systems of classification. When compared to the freestyle (the most popular swim stroke), the panel reached consensus (79%) after the second survey that the differences in the visual information relied on in the *breaststroke* were too small to warrant a specific system of classification for that stroke. Similarly, after the third survey the majority of the panel felt that stroke-specific classification was not warranted for the *butterfly* (71%) or the *backstroke* (57%), though the panel did not strictly reach consensus on either point. In particular, comments were made that the nature of the visual information in the backstroke may be considerably different to that of the other three strokes, given that the swimmer faces upwards in the backstroke. Given the lack of consensus for the butterfly and backstroke, there is reason for evidence to be collected in an experimental study to compare the impairment-performance relationship across these different strokes.

When asked about the distances of the races in comparison to the 100 m race, there was consensus after the second survey that any difference in the visual information relied on in the 50 m (79%) and 200 m (93%) events did *not* warrant specific classification systems.

### Section 8: Congenital and Acquired Vision Impairments

At present, swimmers with comparable levels of VI compete against each other in the same sport class, irrespective of the age at which they acquired their impairment. For swimming, our group of experts almost unanimously agreed (93%) that the age at which an athlete acquired their VI *does* influence the impact of the impairment on swimming performance. Given a scenario where two swimmers had the same level of VI, but one had a congenital impairment from birth and the other an impairment acquired during adulthood, the panel unanimously agreed (100%) after the first survey that the swimmer with the impairment from birth would have a significant disadvantage. The panel’s main comment was that having had sight previously makes it easier for a swimmer to have acquired general motor skills as well as to have learned swimming techniques, which puts them at an advantage when compared to the swimmer who had their VI from birth.

To further explore this issue, in the second survey we sought to establish whether the impact of a congenital (vs. acquired) impairment would differ for swimmers who were completely blind, and for those who had some remaining vision. There was unanimous agreement (100%) that the age at which the impairment was acquired would influence the outcome of competition for swimmers who were completely blind, while the panel was less convinced (71% agreement) for swimmers with some remaining vision.

When asked whether the age at which VI is acquired should be accounted for during classification, the previous panel of experts *across all VI sports* failed to reach consensus (44%), largely because it was felt that the benefits of doing so would be outweighed by the complexity it would add to classification (Ravensbergen et al., 2016). The panel in this study somewhat disagreed with this view when applied to swimming. The panel was presented with a scenario where they were asked to assume that evidence did exist which showed that swimmers who acquired their impairment at a young age had a significant disadvantage in their ability to develop skill in swimming due to an inability to observe others when learning to swim. The panel reached consensus (75%) that if this were to be true, then the benefits of allowing for the age of acquisition of their impairment during classification would outweigh the complexity that doing so would add to classification. However more broadly, even after the third survey, the panel were still undecided whether classification should indeed account for the age at which VI was acquired. For athletes who are completely blind, the panel did not reach consensus (57% agreement) on whether the age of acquisition should be taken into consideration during classification. Similarly, the panel did not reach consensus when asked the same question for athletes with some remaining vision (43% agreement; **Supplementary Table 2**).

### Section 9: The Use of Blackened Goggles

Swimmers with vision impairment who compete in the S11 class (i.e., those with the most severe VI) are required to wear blackened goggles during all events, irrespective of whether they do or do not have any remaining vision. The rationale behind this rule is to ensure that all swimmers are effectively blind when competing. Ravensbergen et al. (2016) found that blindfolds that completely occlude vision are generally inappropriate as an approach to minimize the impact of impairment on the outcome of competition because they prevent athletes from making use of the limited vision that they do have, and instead increase their level of VI during competition. However, they also found that there were some situations in which it is acceptable to use blindfolds.

In our study, the panel was asked on their views about the appropriateness of the use of blindfolds in VI swimming. There was consensus (87%) after the first survey that the use of blackened goggles is a fair way to equalize the impact of impairment on performance in the S11 class (i.e., equalizing those with potentially some and those certainly without any functional vision). However, they also agreed (93%) after the first survey that requiring the use of blackened goggles for *all* VI swimmers (i.e., with all VI swimmers competing against each other while wearing blackened goggles) would *not* create fairer competition.

## Discussion

The aim of this study was to establish expert guidance on the specific requirements for a sport-specific system of classification for VI swimming. To do so we particularly focused on reaching expert consensus on (1) which aspects of visual function are most likely to be related to swimming performance, (2) what specific components of the swimming race are most likely to be impacted by VI, (3) how to handle practical and procedural issues such as testing vision with the best possible optical correction within classification for VI swimming, and (4) other issues particular to VI sports that were identified to require sport-specific decisions (e.g., the use of blindfolds, or whether classification should account for the age at which an athlete acquired their impairment).

We consulted a panel of 16 experts in VI swimming using the Delphi approach to reach consensus on issues that help to guide the development of an evidence-based system. The panel agreed that the system currently used for the classification of VI swimmers does not fulfill the IPC’s aim to minimize the impact of impairment on the outcome of competition. This indicates a clear desire for changes to be made to the way that VI swimmers are presently classified. The results of the surveys help to guide the type of vision tests required for classification, the conditions in which they should be tested, and the particular aspects of swimming likely to be impacted by an impairment to different aspects of vision.

### Aspects of Vision That May Impact Swimming Performance

An important goal of this study was to identify those aspects of visual function that are not presently tested during classification, but may be related to performance in swimming. The panel highlighted two measures of visual function, depth perception and light sensitivity, which they believed were important enough for swimming performance to be considered for inclusion in classification. In addition, a further two measures, contrast sensitivity and motion perception, did not strictly reach consensus, yet may be worthy of further investigation given the strong support they received.

In other areas of research interested in the effects of vision impairment, it has recently been shown that more specific aspects of visual function are better at predicting functional abilities. For example, contrast sensitivity has been shown to better predict driving performance than visual acuity or visual field (Wood et al., 2013), and it is also associated with impairments in gait when visual acuity is not (Duggan et al., 2017). This aligns with our expert panel’s suggestion that specific aspects of visual function such as depth perception, light sensitivity, contrast sensitivity, and motion perception are likely to be predictive of swimming performance.

### Specific Components of the Swimming Race Affected by VI

The second key goal of this study was to identify the specific components of a swimming race that are most likely to be negatively affected by the presence of impaired vision. The results led to the development of a model (**Figure 1**) that outlines the nature of the changes in performance that should be expected with increases in VI. The panel indicated that VI does not impact one particular part of the swimming race exclusively, but rather they identified aspects of performance in each of the four segments (i.e., start, clean swim, turn, and finish) of the race that are likely affected by impairments in visual function.

Many of the race components identified by the panel as being likely to be affected by VI are not normally thought of as performance parameters in sighted swimmers, but may prove to be useful parameters for assessing the performance of VI swimmers. For example, the ability to navigate within a lane is unlikely to be an important predictor of race outcome in sighted swimmers, yet may be an important determinant of performance in an S11 race, where swimmers are often observed to either swim very close to or even touch the lane rope for navigation, or zigzag across the width of the lane. Similarly, using the allowed length of the underwater phase (i.e., 15 m) both after the start and the turn is not usually assessed in sighted swimmers, even though there is evidence that using the maximal allowed underwater distance is related to better times (Cossor and Mason, 2001; Mason and Cossor, 2001). According to the experts, VI swimmers often choose to come to the surface earlier to minimize the risk that they deviate into the lane beside them, which would in turn negatively impact their overall swimming performance.

In contrast, for a number of the well-established parameters used to analyze swimming performance (e.g., stroke length, and stroke rate) the panel agreed that they would *not be affected by* VI, or in some cases (e.g., reaction time to the start signal) VI might even provide a slight advantage. This is consistent with findings from previous studies which show that stroke rate and stroke length do not differ across the three VI classes (Malone et al., 2001; Burkett and Mellifont, 2008; Daly et al., 2009). This finding that the typical determinants of swimming performance would differ to those necessary to evaluate performance in VI swimming clearly highlights the need to consult with experts in the field to identify the determinants of sport performance likely to be affected by the impairment of interest.

### Including the Age at Which VI Is Acquired in Classification

The panel was very clear in their opinion that the age at which an athlete acquired their impairment has a significant impact on swimming performance. Specifically, they agreed unanimously that a person who had acquired their impairment later in life has an advantage over someone who has the same level of vision but had lost it from birth. From the expert consultation performed across all VI sports, it was clear that the impact of the age of acquisition on performance is likely to differ largely on the basis of the complexity of the motor skills required for that sport (Ravensbergen et al., 2016). Generally, in sports that rely on more complex motor skills, those with an acquired impairment were believed to possess an advantage over those who have a congenital impairment (Ravensbergen et al., 2016). However, the advantage was deemed to be less likely to exist in sports where the movement form is more simple (e.g., rowing or cycling). This aligns with knowledge in the area of motor development which suggests that vision is likely to play a central role in the acquisition of many motor skills. Indeed it is still possible to learn motor skills in the absence of vision, but it is likely that the rate of learning would be lower in the absence of vision. For instance, blindness would prevent the ability to exploit observational learning to model movements on those of others (Hodges and Williams, 2007; Hodges et al., 2007), and would prevent the use of the mirror neuron system whereby motor learning can be enhanced though observation (Logo-Rodriguez et al., 2014). Because swimming is a sport that requires athletes to master complex motor skills to reach the elite level, this outcome could be expected. A number of panelists raised the case of a specific swimmer who had been a top-performing S11 swimmer who was a competitive able-sighted swimmer who lost their sight in their mid-20s. The panel felt that swimmer’s dominance in the S11 class suggests that swimmers who are severely visually impaired from birth are at a disadvantage because they did not have the opportunity to use visual information when developing their swimming skills.

Nonetheless, the panel remained undecided whether a future VI classification system should indeed account for the age at which the impairment was acquired. Only in the scenario that there was clear evidence to show that swimmers who acquired their impairment at a very young age are at a clear disadvantage in the ability to acquire elite swimming skills, the panel agreed that the benefits of accounting for the age of acquisition in classification outweighs the added complexity to VI classification of doing so. It clearly is an important issue for VI classification in swimming and further classification research should be conducted to provide the necessary evidence about its true effect on swimming performance to be able to make an evidence-based decision about whether to account for it in future classification.

### The Use of Blindfolds

The panel was very clear on the topic of blindfolds - they agreed that the current rule requiring S11 athletes to wear blackened goggles is appropriate to provide fairer competition, but that it would be inappropriate to require all VI swimmers to wear blackened goggles. Although these results are clear, the question remains whether those who currently compete in the S11 class but do have some remaining vision could actually use that remaining vision to facilitate their performance in the pool. This should be empirically verified in future work to establish whether those individuals belong in the same class as those who are completely blind.

### The Panel

Our expert panel was designed to provide a valid representation of the views of athletes, coaches, administrators, and scientists involved with VI swimming. Of course there is a degree of subjectivity when selecting panel members, particularly when it is difficult to define the concept of expertise in the sport. Fortunately, we were able to work closely with the International Paralympic Committee and World Para Swimming to identify people who were considered to possess specific expertise in the sport. This maximized the chance that we could identify and consult with the best qualified people involved with the sport. Given that the primary aim of the study was to establish expert guidance for a sport-specific classification system for VI swimming, the primary goal of the study was to canvas the viewpoints and concerns of a sufficiently representative sample of individuals from the sport. In this sense we were less concerned with reaching consensus on topics, but rather to provide a framework from which research can be performed to, in many cases, provide empirical evidence to support or refute the views of the expert panel.

### Implications

The results from this expert consultation have delivered clear guidance to develop a theoretical model of how VI might impact performance in swimming. In particular, recommendations have been provided for the types of visual functions that are not currently tested during classification but should be considered for inclusion in a future system, and the key components of a swimming race that might be affected due to impairments in vision. Also, the panel provided clear suggestions for how to handle practical and procedural issues around classification of VI for swimming. Overall these results provide clear guidance for the design of empirical studies that seek to investigate the relationship between VI and performance in swimming.

Combining the results from this future empirical studies with the experts’ suggestions for how to handle the major practical and procedural issues will together provide the necessary tools to develop an evidence-based VI classification system specifically designed for the sport of swimming. This will be a major step toward enhancing the legitimacy of VI sports in the sense that the winner should be the best athlete instead of the athlete with the least severe VI. Realizing this important step will not only create fairer competition at the elite level, but additionally is likely to increase participation by VI athletes across all competition levels within the sport of swimming.

## Author Contributions

HR contributed to the study design, survey development, data analysis phases of this manuscript, wrote the draft of the manuscript, and revised the manuscript according to the co-authors’ comments and suggestions. AG contributed to the study design, survey development, data collection, data analysis phases of this manuscript, and provided comments and suggestions to the draft versions of the manuscript. DM contributed to the study design, survey development phases of this manuscript, and edited the draft versions of the manuscript.

## Conflict of Interest Statement

The authors declare that the research was conducted in the absence of any commercial or financial relationships that could be construed as a potential conflict of interest.

## Footnotes

1 Athletes can be classified based on two measures of visual function: (*i*) visual acuity, a measure of the sharpness of central vision, and (*ii*) visual field, a measure of the size of the area which is seen. An athlete is allocated class S13 when they meet at least the minimum impairment criteria, which for visual acuity currently is 1.0 logMAR, and visual acuity is up to 1.4 logMAR, or their visual field is at least restricted to 40 degrees diameter. An athlete is allocated class S12 when their visual acuity lies between 1.5 and 2.6 logMAR, or their visual field is restricted to <10 degrees diameter. Athletes can only be allocated the S11 class based on their visual acuity, which needs to be worse than 2.6 logMAR.
