# Supplementary material for: Expert Consensus to Guide the Classification of Paralympic Swimmers With Vision Impairment: A Delphi Study
Source: Front Psychol. 2018 Oct 17;9:1756. doi: 10.3389/fpsyg.2018.01756 (PMC6199393; doi:10.3389/fpsyg.2018.01756)
Supplement: Supplementary file 1 [file Table_1.docx]

**Supplemental table 1**. Process of reaching consensus on the central questions in each section of the Delphi review.

| Section | Central question(s) | Result/action after survey 1 | Result/action after survey 2 | Result after survey 3 |
| --- | --- | --- | --- | --- |
| 1. Aim of classification | Does the current system classification system within VI swimming fulfil the aim to minimise the impact of impairment on the outcome of competition? | **Consensus reached** |  |  |
| 2. Minimum impairment criteria | Is the current minimum impairment criterion for **visual acuity** set appropriately for VI swimming? | **Consensus reached** |  |  |
|  | Is the current minimum impairment criterion for **visual field** set appropriately for VI swimming? | **Consensus reached** |  |  |
| 3. Sport classes | Do the levels of impairment **within** a single class have a reasonably comparable impact on swimming performance? | **Consensus reached for S11** |  |  |
|  |  | Concerns addressed for S12 and S13 classes | Questions clarified and rephrased | No consensus reached |
|  | Do the levels of impairment **across** two separate classes have a considerably different impact on swimming performance? | **Consensus reached for S11/S12 border** |  |  |
|  |  | Question rephrased for S12/S13 border | Question redirected: *what are the most appropriate number of classes?* | No consensus reached |
| 4. Measures of visual function* | Which aspects of visual function are most likely to affect swimming performance? | **Consensus reached for visual acuity and visual field** |  |  |
|  |  | List of aspects of visual function created and put forward | **Consensus reached** |  |
| 5. Procedures for testing visual function during classification | Should classification be based on the results of the best eye? | Concerns addressed | Question rephrased | Nearly consensus |
|  | Should vision be assessed while wearing the best possible correction? | Concerns addressed | **Consensus reached** |  |
| 6. Impact of VI on specific components of a swimming race * | Which aspects of swimming performance are most likely to be impacted by vision impairment? | List of aspects of performance created and put forward | **Consensus reached** |  |
| 7. Impact of VI on different strokes and distances | Does the impact of vision impairment differ across the different swimming strokes? | Concerns addressed | Question rephrased | Nearly consensus |
|  | Does the impact of vision impairment differ across the different swimming distances? | Question rephrased | **Consensus reached** |  |
| 8. Congenital and acquired vision impairments | Does the age at which the impairment was acquired affect the impact is has on swimming performance? | **General consensus reached** |  |  |
|  |  | Further exploration for subgroups:  (1) those who are completely blind, and (2) those with some remaining vision | **Consensus reached for subgroup 1** |  |
|  |  |  | Nearly consensus for subgroup 2 |  |
|  | Should classification account for the age at which the impairment was acquired? | Further exploration for subgroups:  (1) those who are completely blind, and (2) those with some remaining vision | Questions rephrased | No consensus |
| 9. The use of blackened goggles and tappers | Does the use of blackened goggles provide a fair way of equalising the impact of vision impairment on swimming performance? | **Consensus reached** |  |  |
|  | Would having a tapper in place for all swimmers equalise the impact vision impairment has on the turn and the finish for all VI classes? | Consensus reached |  |  |
