# Supplementary material for: Expert Consensus to Guide the Classification of Paralympic Swimmers With Vision Impairment: A Delphi Study
Source: Front Psychol. 2018 Oct 17;9:1756. doi: 10.3389/fpsyg.2018.01756 (PMC6199393; doi:10.3389/fpsyg.2018.01756)
Supplement: Supplementary file 2 [file Table_2.docx]

**Supplemental table 2**. Final questions posed in each of the nine sections of the Delphi review.

| Topic | Questions | Answers: # (*% after exclusion*) |
| --- | --- | --- |
| 1. Aim of classification | Do you believe the way that vision impairment is currently classified within IPC Swimming fulfils the aim to minimise the impact of the eligible impairment on the outcome of competition? | Yes: **2 (13*%*)**  No: **5 (*33%*)**  Partially: **8 (*53%*)**  I don’t feel qualified: **1 (a coach)** |
| 2. Minimum impairment criteria | Do you believe that the current minimum impairment criterion for **visual acuity** accurately represents the least severe level of vision impairment that would decrease swimming performance*?* | Yes: **10 (*83%*)**  No: **2 (*17%*)**  I don’t feel qualified: **4 (2 athletes, 1 coach, 1 scientist)** |
|  | Do you believe that the current minimum impairment criterion for **visual field** accurately represents the least severe level of vision impairment that would decrease swimming performance*?* | Yes: **10 (*83%*)**  No: **2 (*17%*)**  I don’t feel qualified: **4 (1 athlete, 2 coaches, 1 scientist** |
| 3. Sport classes | If the swimmers in the S11 class were to compete against the S12 swimmers, do you believe that the swimmers in the S11 class would be at a disadvantage when compared to the swimmers in the S12 class? | Yes: **15 (*100%*)**  No: **0 (*0%*)**  I don’t feel qualified: **0** |
|  | Do you believe that the level of vision impairment of the swimmers **within the S11** class have a reasonably comparable impact on swimming performance? | Yes: **12 (*80%*)**  No: **3 (2*0%*)**  I don’t feel qualified: **1 (an athlete)** |
|  | If the swimmers in the S12 class were to compete against the S13 swimmers, do you believe that the swimmers in the S12 class would be at a disadvantage when compared to the swimmers in the S13 class? | Yes: **11 (69*%*)**  No: **5 (*31%*)**  I don’t feel qualified: **0** |
|  | Is the range of severity of the vision impairments within the **S13** class appropriate to provide equal competition within this class?  I.e., can the swimmers with the most severe vision impairment in this class compete equally against those with the least severe visually impaired swimmers of this class? | Yes: **5 (*36%*)**  No: **9 (*64%*)**  I don’t feel qualified: **1 (an athlete)** |
|  | Is the range of severity of the vision impairments within **the S12 class** appropriate to provide equal competition within this class?  I.e., can the swimmers with the most severe vision impairment in this class compete equally against those with the least severe visually impaired swimmers of this class? | Yes: **9 (64%)**  No: **5 (35%)**  I don’t feel qualified: **1 (an athlete)** |
|  | Do you believe that **the number of classes** currently used is appropriate to fulfil the aim of classification? | Yes: **6 (*46%*)**  No: **7 (54*%*)**  I don’t feel qualified: **1 (an athlete)** |
|  | If not, how many classes do you believe would be most appropriate? ‡ | Two: **4 (*67%*)**  Four: **2 (33*%*)**  Other, namely … : **1 (an athlete)**  I don’t feel qualified: **0** |
|  | Do you believe that **the number of VI classes should be minimised** to ensure there is a sufficient number of swimmers in each class? | Yes: **6 (*43%*)**  No: **8 (57*%*)**  I don’t feel qualified: **1 (round 3)** |
|  | Do you believe that **the range of impairment severity** in each VI class should be **minimised** to ensure equal competition between the swimmers? | Yes: **9 (*75%*)**  No: **3 (25*%*)**  I don’t feel qualified: **3 (round 3)** |
| 4. Measures of visual function* | Do you believe that the assessment of **visual acuity** provides an appropriate way to assess the impact of VI on swimming performance? | Yes: **11 (*79%*)**  No: **3 (*21%*)**  I don’t feel qualified: **2 (1 athlete, 1 coach)** |
|  | Do you believe that the assessment of **visual field** provides an appropriate way to assess the impact of VI on swimming performance? | Yes: **11 (*94%*)**  No: **3 (*6%*)**  I don’t feel qualified: **2 (1 athlete, 1 coach)** |
|  | Do you believe that the assessment of **only** visual acuity and visual field is sufficient for the classification of swimmers with vision impairment? | Yes: **1 (*7%*)**  No: **11 (*93%*)**  I don’t feel qualified: **0** |
| 5. Procedures for testing visual function during classification | Do you believe the current practice of classification based on results of the best eye alone is the most appropriate approach? | Yes: **4 (*29%*)**  No, should be both eyes: **10 (*71%*)**  I don’t feel qualified: **1 (a coach)** |
|  | Which of the following do you believe to be the most appropriate approach to use for the classification of athletes with vision impairment? | Using the best possible optical correction, irrespective of whether it can be worn in the pool: **10 (83*%*)**  Using the best possible optical correction that the classifier agrees can reasonably be worn in the pool: **2 (*17%*)**  I don’t feel qualified: **2 (1 athlete, 1 coach)** |
| 6. Impact of VI on specific components of a swimming race * |  |  |
| 7. Impact of VI on different strokes and distances | Do you believe that the difference in the nature of the visual information relied on during **breaststroke** (when compared to freestyle) warrants specific classification criteria for breaststroke? | Yes: **3 (*21%*)**  No: **11 (*79%*)**  I don’t feel qualified: **0** |
|  | Do you believe that the difference in the nature of the visual information relied on during **butterfly** (when compared to freestyle) warrants specific classification criteria for butterfly? | Yes: **4 (*29%*)**  No: **10 (*71%*)**  I don’t feel qualified: **0** |
|  | Do you believe that the differences in the nature of the visual information relied on during **backstroke**, when comparing to the freestyle, justifies specific classification criteria? | Yes: **6 (*43%*)**  No: **8 (*57%*)**  I don’t feel qualified: **0** |
|  | Do you believe the difference in the nature of the visual information used during the **50 metre race** (when compared to the **100 metre** race) warrants specific classification criteria for the 50 metre race? | Yes: **3 (*21%*)**  No: **11 (*79%*)**  I don’t feel qualified: **0** |
|  | Do you believe the difference in the nature of the visual information used during the **200 smetre race** (when compared to the **100 metre** race) warrants specific classification criteria for the 200 metre race? | Yes: **1 (*7%*)**  No: **13 (*93%*)**  I don’t feel qualified: **0** |
| 8. Congenital and acquired vision impairments | For an athlete with vision impairment, do you believe that the age at which the impairment was acquired influences the impact of the impairment on swimming performance? | Yes: **14 (*93%*)**  No: **1 (*7%*)**  I don’t feel qualified: **1 (a scientist)** |
|  | Imagine a scenario where two swimmers have the same level of vision impairment, but they differ in the age at which the impairment was acquired competed against each other. One athlete has a congenital impairment (present at birth) and the other an acquired impairment (developed as an adult).  *Which of the following statements do you agree with the most?* | The swimmer with the congenital impairment is at a disadvantage: **12 (*100%*)**  The swimmer with the acquired impairment is at a disadvantage: **0**  The impact of the impairment should be the same for the two swimmers: **0**  I don’t feel qualified: **4 (1 athlete, 3 coaches)** |
|  | If classification were to take into account the age at which the impairment was acquired, which of the following options do you believe is the most appropriate? | Swimmers with congenital and acquired impairments should compete in the same class, but different thresholds should apply depending on the age at which the impairment was acquired: **5 (83%)**  There should be separate classes for swimmers with congenital and acquired impairments: **1 (17*%*)**  I don’t feel qualified: **9 (4 athletes, 4 coaches, 1 scientist)** |
|  | Imagine that evidence shows that the age that swimmers acquire their impairment significantly impacts their ability to acquire skill in swimming.  Do you believe that the benefits of accounting for the age that a swimmer acquired their impairment would outweigh the added complexity in classification for swimming? | Yes: **9 (*75%*)**  No: **3 (*25%*)**  I don’t feel qualified: **3 (1 athlete, 1 coach, 1 scientist)** |
|  | For athletes **who are completely blind**, do you believe the age at which the impairment was acquired will influence how much the impairment impacts their swimming performance? | Yes: **14 (*100%*)**  No: **0**  I don’t feel qualified: **0** |
|  | For athletes **who are completely blind**, do you believe the age at which the impairment was acquired should be taken into account in classification? | Yes: **8 (*57%*)**  No: **6 (*43%*)**  I don’t feel qualified: **0** |
|  | For athletes **who have some remaining vision**, do you believe the age at which the impairment was acquired will influence how much the impairment impacts their swimming performance? | Yes: **10 (*71%*)**  No: **4 (*29%*)**  I don’t feel qualified: **0** |
|  | For athletes **who have some remaining vision**, do you believe the age at which the impairment was acquired should be taken into account in classification? | Yes: **6 (*43%*)**  No: **8 (*57%*)**  I don’t feel qualified: **0** |
| 9. The use of blackened goggles and tappers | Do you believe that the use of blackened goggles is a fair way of equalising the impact of impairment on performance in the S11 class (i.e., to equalise those with some and those with no functional vision)? | Yes: **13 (*87%*)**  No: **2 (*13%*)**  I don’t feel qualified: **0** |
|  | Do you believe that requiring the use of blackened goggles for **all VI swimmers**, so there is only one VI class, would create a fair competition? | Yes: **1 (*7%*)**  No: **13 (*93%*)**  I don’t feel qualified: **1 (a coach)** |
|  | Would having a tapper in place for all swimmers equalise the impact vision impairment has on the turn and the finish for all VI classes? | Yes: **3 (*20%*)**  No: **12 (*80%*)**  I don’t feel qualified: **1 (an athlete)** |

* Follow-up questions on potential additional measures of visual function and impact of specific components of a swimming race can be found in Tables 2 and 3

‡ Question only presented to panelists who answered ‘no’ to the previous question.
