# Supplementary material for: Expert Consensus to Guide the Classification of Paralympic Swimmers With Vision Impairment: A Delphi Study
Source: Front Psychol. 2018 Oct 17;9:1756. doi: 10.3389/fpsyg.2018.01756 (PMC6199393; doi:10.3389/fpsyg.2018.01756)
Supplement: Supplementary file 3 [file Table_3.docx]

**Supplemental Table 3**. List of measures of visual function and their definition as provided to the panel.

| **Visual function** | **Description** |
| --- | --- |
| Visual acuity | The clarity of vision. |
| Visual field | The peripheral field of view someone has without moving their eyes |
| Dynamic visual acuity | The ability to clearly see a moving target |
| Ocular coordination | The ability of both eyes to move together |
| Depth perception | The ability to see the world in three dimensions (e.g. to estimate the distance to an object) |
| Motion perception | The ability to estimate the speed and the direction of a moving object |
| Contrast sensitivity | The ability to distinguish objects from their background |
| Light sensitivity | The impact of bright light on the ability to see clearly |
